# Working in the wake: transformative global health in an imperfect world

**DOI:** 10.1136/bmjgh-2022-010520

**Published:** 2022-09-19

**Authors:** Rochelle A Burgess

**Affiliations:** 1 Institute for Global Health, University College London (UCL), London, UK; 2 Department of Social Work, University of Johannesburg, Auckland Park, South Africa

**Keywords:** Health services research, Public Health

## The beginning: long ago, is now

The problems with global health are many. They are as old as the discipline itself, inherited from colonial projects and paradigms that have existed as long as there has been exploration into other lands. In her recent work *In the Wake: On Blackness and Being*, Christina Sharpe^1^ asks us to imagine, and acknowledge, that the world and life of Black people, exist in and are shaped by the enduring afterlife of slavery, colonialism and racism. This moment in global health is highly attuned to this fact. There is an ‘awakening’ to the fact that every dimension of global health praxis has been inexorably shaped by its past. Undoubtedly, there has been an underappreciation of the consequences of the productive power of the ever-expanding body of knowledge, praxis and practice that determines the why, what and how of our discipline. This is the power of what historians dub the archive—the collection of records, documents and evidence that define an institution. And our archive has locked us into something we desperately need to change.

A glimpse into the archive reminds us of the longevity of global health’s problems that have been faced by every generation, often hindering efforts to improve the health of others. For example, in 1978, global powers had the opportunity to institute and fund a health systems model founded in the scaling up of local power, resources and capacity, in the form of primary healthcare.^2^ The wide sweeping changes were a response to calls for social justice from millions of people in resource poor settings driven to their position through the extractive policies of colonialism, expansionism and cold war politics. Alma Ata and its 134 signatories were called to build opportunities for communities to build health enabling environments as part of eradicating illness. Almost immediately, powerful countries funded activities totally counter to these principles—choosing instead to focus on single diseases. This ‘selective’ primary healthcare^2^ was a hunt for magic bullet cures linked to technological advances owned by rich countries. More than forty years later, we are still on a quest for ‘health for all’, working our way through ongoing waves of crisis, which can at times feel seemingly impossible to overcome in the absence of truly radical change. This is a clear example of the reality of working in the wake in global health; our efforts to change health are hindered by the power of the few. And the longstanding hesitancy to acknowledge the worst of this ‘wake’ means it will likely always be with us.

As a field we readily acknowledge the need for change. Change in the way we fund and structure our institutions,^3^ change in the way we educate current and future generations,^4^ change in the way we produce knowledge.^5^ All these changes draw our focus to the necessity of a deeper and more nuanced understanding of one concept: power. Despite being presented as a singular term, it is plural and intersecting by nature, ever present, ever working, chronically underestimated and frankly oversimplified within our projects of change in the global health industry. To move this discipline forward demands a turning towards this complexity, in search of new tools to embrace, respond and work alongside it. To do so, we require methods that allow us to work in the past, present and future simultaneously. We need research governed by logics of care that demand the responsibility of actors—specifically researchers, who recognise the *past* at work in the now (see Hirsch’s work^6^). This speaks directly to a longstanding need for our methods and methodologies to mean something to the people—everyday people—with whom this problematic project of global health engages.

However, to occupy positions that truly centre people within our global health frame is so much more complicated than we acknowledge. For better or worse, the past, and present of global health is an interventionist one. As Packard^7^ reminds us in his history of global health, our work always has been project based. Global health actors are propelled by a series of projects, responding to funding calls, which break the complexity of peoples’ daily lives into ‘manageable bits’, and ‘meaningful outcomes’. This simplification means that, after, millions of projects and billions of dollars, gains can quickly dissipate. For example, following huge strides in reduction of child marriages globally—the COVID-19 pandemic resulted in an increase in over 2.5 million child marriages, when economic insecurity was widespread, and marriages emerged as a potential route for resources.^8^

Beyond this, interventionism can also mute the ability of researchers and practitioners to fully understand the complexity of living life in the wake—and its capacity to contain more than suffering. The drive to help also contributes to an erasure of agency, an assumption of the absence of capability, a predisposition to see only deficits where there are strengths. Sharpe describes this as the need to remember that there are always those who find ways to make a path through the wake—an ability to make ”liveable moments, spaces and places in the midst of all that was unliveable”. (1, p4)

But must it be this way? Perhaps, as we look for new ways to ‘fix’ the discipline, we can lean into the complexity of the wake, and find a way to accept the uncomfortable path which works in search of social change in true partnership with the voices of everyday people.

Weick^9^ wrote about the challenge of social change—noting our limited psychological capacity to comprehend and act in response to huge systems when overwhelmed. He suggests that projects of social change are difficult to deal with because they are so huge in scope. That they activate a series of responses that make action difficult. In response, he argues that actors supporting projects of change are better off accepting two realities : (1) that change happens in stages, building on small acts, contributing to a larger project of improvement and (2) the importance of these small acts being tangible, and ultimately meaningful in the short and long term, calling us:

*To recast larger problems into smaller, less arousing problems, [so] people can identify a series of controllable opportunities of modest size that produce visible results and that can be gathered into synoptic solutions. This strategy of small wins addresses social problems by working directly on their construction and indirectly on their resolution. Problems are [re] constructed to stabilize arousal at moderate intensities where its contribution to performance of complex tasks is most beneficial (9, p40).*

This is important for global health in two ways. First, it allows us to seek to understand problems as a sum of their parts—and highlights the value of work towards change, in tackling some of the parts. The second contribution that this makes to global health is his call also prevents us from being satisfied with the intervention in and of itself. Projects should be seen as contributing ‘*indirectly to their solution’*—an acknowledgement of the longer term processes that require slow work, which contribute overtime to chipping away at the enormity of the social problem.

Think for a moment of the rise in popularity and importance of theories of change in global health work. This method is a direct response to the enormity of problems; to break them into smaller parts and processes. The result, however, has been to build interventions that rarely appear as complex as the challenge that faces us. We push the most embedded and complex realities beyond our ceilings of accountability—washing our hands of the very contexts that drive the manifestation of health challenges that scholars, practitioners and beyond claim to be committed to changing. We do this in part because we have to—the enormity of the wake is almost impossible to bear at times.

As a result, it is the second part of Weick’s call which presents the largest hurdle for researchers. We struggle to see the long arch of the solution, how each project has the potential to contribute to the larger goal of change in people’s lives in the long *and* short term. Institutions are not designed to enable us to work in these ways simultaneously. The compartmentalisation and discursive barriers we live and work through leave us blind not only to the complexity of the longer term goal but also to the short-term real-world impacts that citizens rightly demand of us along the way. We design interventions about mental health improvement—but cannot grapple with what it means when people say their depression is caused by hunger—because that is outside our disciplinary domains. We struggle to take the reality of the wake seriously because the actors who make decisions about what projects should be taken on, what should be included and what long-term challenge we are working towards solving—continually exclude those living in the wake of these processes.

Despite the extensive application of participation, citizen science and community involvement, our methods have not saved us, because methods alone do not ask us to generate real-time impacts in the lives of those we encounter. The process and ownership of dissecting the larger problem has contributed to a moving away from the people who live these problems everyday, who live in the wake of our horrible history. We cannot deliver logics of care which fully hear what is being asked of us, because we are not set up to listen. Our work has fragmented us to the point that we rarely see the fullness of the lives of those we seek to support. Or if we do, we are bound by the logics of institutions that continue to benefit from the wake, pulling us in other directions. We seem to have collectively lost who we are here for, and what we are working towards.

## The middle: who are we writing/working for?

*What happens when we look at and listen to these and other Black girls across time? What is made in our encounters with them? (Sharpe, 2016 p51).*

*When you called, I thought it was the electricity company coming to give me electricity, because that’s what I told the last researcher who was here that I needed—(Bumi, KZN 2010).*

Despite the claims to service social and global justice within our field, global health often feels as though it has long forgotten *who* it is for. Our debates on inclusion of local voices typically focus on researchers and academics.^10^ While this is important, researchers from low resource settings and minoritised backgrounds may still occupy social positions that distance them from the starkest realities of life in the wake as well. They live with relative advantage compared with the people who their projects are about. What is more, for them to participate in the process of expanding the global health canon, they must transform themselves into that canon,^11^ potentially muting the disruptive power they hold.

One way to overcome this distance is to apply methodologies that create a greater proximity to the experiences of others. In many instances, qualitative and participatory methods (which I use almost exclusively) are positioned as superior in this regard, as they allow participants to ‘speak for themselves’. However, during my doctorate, when Bumi said the above quote to me as I left her home after an afternoon spent completing a life history interview, it became painfully clear that documenting stories in the hopes that they may bring about change does not manifest through the telling of stories alone.

When stories are told collected and translated into ‘evidence’ they enter spaces that are not neutral. The qualitative canon may seem willing, but it too is largely incapable of hearing them. The act of hearing is impossible as stories themselves become twisted, shaped and transformed by the discourses they encounter, moving them away from the desires and aims of those we initially sought to help and whose voices we aimed to elevate. Janet Stoppard’s^12^ work on the social construction of women’s depression grasps this beautifully; ‘*for the ability for women’s concerns to be heard, they must be packaged in the language of science; but as soon as that language is spoken, any attempt at critique of its limitations, is weakened’* (pg 98). This is true for all actors on the receiving end of global health, and development—and is what Indigenous, critical and decolonial thought has been trying to articulate for some time^13 14^. Global health struggles to acknowledge that people do not simply, or only live in subjection and as the subjected (1, p4). They aspire, they continally engage in projects of change—their very survival is a manifestation of this. And as such, their voices carry truths that must be held and centred in projects of change designed on their behalf.

This is a critical acknowledgement, as it reminds us that methods don’t automaticlaly demand a holding or centering of truths. This is because the global health enterprise is inextricably bound up in the performativity of the existing canon. The erasure of everyday people, their perspectives and voices, remains a central pillar of a postcolonial project, as it was a within colonial ones, because it struggles to hold hear a truth without its transformation. In global health this erasure feels systematic, enabled by proxy of science, and the evidenced-based paradigms we serve. What is sayable, who can say it, and how it must be said for it to fit into the Archive is largely fixed. As noted recently by Abimbola^15^ in global health, this privileging of the voices of thinkers (*Professors*), over doers (*Plumbers*), has been unjust. Redressing these injustices will not be helped simply through new methods of documentation (as called for recently by Topp and colleagues^16^ in seeking understand power at work in global health). Nor will it automatically be helped by the necessary calls for diversifying the global health work force. None of these things matter if the tools we use continue to pull us away from the complexity of people’s truths.

But what is to be done with this? Is there a form of global health research that can hear and act on what is asked of us? In making sense of this in my own work, I often start with a single orienting question. *What does my work leave behind?* I have asked this question, for the past decade because of that meeting with Bumi—an encounter that showed me the near impossibility of avoiding violence with a western scholarship predisposed to silencing. Can we conduct our work, so that it leaves us in a place where the bodies and voices who call to us throughout time, have some form of recourse in the here and now?

## The end (and to begin again): long live transformation

*This looking makes ethical demands on the viewer; demands us to imagine otherwise; to reckon with the fact that the archive, too, is invention*—Sharpe, 2015, pg 52.

If our archive is invention—then we can reinvent it. Our desire to do so in global health (and elsewhere) has led to the prioritisation of ‘participant and patient involvement’. The obsession with these terms and the wider umbrella of participation is longstanding. But there is limited acknowledgement of the political and power dimensions at work within participatory approaches. Recently, along with colleagues,^17^ we reviewed definitions of participation used within the development and global health landscape. We identified that most paradigms view participation as working to contribute to change through process, or outcome. To counter this, we drew on the work of White^18^ whose analyses of the failures of participation identify that participation with transformative goals—where it is both the means, and end goal of an intervention—carries the ability to offer a counter response to the erasure enabled by much of the current global health cannon. She defines transformative participation as:

*the practical experience of being involved in considering options, making decisions, and taking collective action to fight injustice (which) is itself transformative. It leads on to greater consciousness of what makes and keeps people poor, and greater confidence in their ability to make a difference (*pg 59).

Critically, transformative participation is not only about changing the ‘researched’ but also changing the ‘researcher’. Illuminating that in order to remove the binaries between varied actors and knowledge producers across global health landscapes, it must be our goal to do this from the start of the encounter.

If global health now finds itself at a stage where we are ready to admit the problematic nature of our archive; to acknowledge its systematic erasure of the very people who the field is supposed to be about, what comes next? What is the work needed to reimagine, reinvent, and build a new archive? How do we do this practically, in a way that allows us to acknowledge that the wider, larger problems of global health may take generations to fix? How do we find routes and opportunities to do this type of work, in ways that refuse to reify the violence of the current state of the archive and our everyday research practices?

There is very little guarantee that participation will be implemented in ways that demand, plan for and facilitate transfers of various forms of power to promote small wins in the lives of everyday citizens. Finding routes and new logics of care is not a question of methods—it is a question of epistemology (what counts as knowledge?) and perhaps more importantly ontology (what is the purpose of global health practice, research and scholarship at all?) among those who currently hold more power in the global health space.

In my work where I remain guided in part by Weick, this manifests in asking myself what opportunities exist for a research project to be transformative in the short (immediate), medium (along the life of the project) and long term (the end goal)? Can transformation fit within the parameters of any given research project?

Recognising the need to work along these three stages is underpinned by a transformative epistemological paradigm, which demands an end to the separation between the types of knowledge actors in global health. If we return to Abimbola’s terminology,^15^ it requires specific work from both *Plumbers* and *Professors*, wherever they are situated in the world. Donna Mertens^19^ defines this paradigm broadly, as an orientation to the purpose and the goal of knowledge production to work from and acknowledge inequality between partners. It is work that is designed to overcome this inequity in the short term (eg, during research) and the long term (through varied projects of change). It demands that we transition from relationships of extraction to relationships designed for the purpose of a transfer of power and resources for both actors, but particularly from professors, to citizens.

However, if we are committed to engageing with what power produces in academia, we must also acknowledge that the ability to drive transformation will not be equally available to all actors in the same way. The academy is filled with scholars on varied career trajectories, working in varied relationships, which enable or hinder safety to make recommendations on the how’s and why’s of research practice. Many minoritised, female and junior scholars lack the safety to overcome their own erasures in the research world. This fact has shaped my own need to settle for small wins and smaller forms of transformation, when the pragmatics of research are determined beyond my decision-making power and safety.

A potential pathway for achieving transformation in various domains is summarised in table 1. It suggests that across any and all methods, there should be at a minimum the use of participatory orientations to design, research questions and the focus of the work as much as possible. The inclusion of remuneration and compensation systems reflect and acknowledge the labour extended by all parties in the production of knowledge and acknowledge the tangible needs as stated by cocollaborators (rather than ‘target groups’). These types of changes are possible within the parameters of any discipline or any modality (quantitative or qualitative) and many are arguments that can be suggested by more positionally vulnerable scholars who have less capacity to shape research they may be involved in but don’t control, to support their desire to serve equity and transformation goals in small ways within the bounds of their work.

**Table 1:** Attempting transformation in (community) global health research

The dream—co-owned research with communities	Where possible—co-design sharing responsibility of decision-making	At a minimum: inclusive methods and acknowledgement of contributions
Co-produced questions and research strategy (priorities and goals are set with the community) Long timelines that allow communities to experience change Transfer of power and resources is the outcome—building foundations for future independent work (if needed) Impact occurs during the research process	Co-design and shared practices of research/project implementation Open and flexible research questions Action to ensure transferability and impact after the study	Participatory methods used where research questions are pre-determined by one group Compensation and partnership systems that reflect understandings of local need Reflection and action on transferability of outcomes.
*Likely actors: senior/protected academics at top of current power structures with flexibility to push boundaries*	*Likely actors: mid-career academics locked within difficult systems, but some control over design*	*Likely actors: early career or precarious researchers with limited control over design*

In an ideal world, all projects would be organised within the paradigm of coproduction. Crucially coproduction should be conducted within a transformative paradigm that acknowledges the differential resource contributions made by all actors. This would ideally come before project planning as a route to making visible, and potentially upsetting and transforming, historical power differentials between groups.^20^ Where possible, projects should attempt to work with communities to establish the boundaries and value of this research to them and their lives.

When using the term community, it must extend beyond the realm of government, policy and NGO stakeholders—to actual everyday people; the *potential service users* of any programme or project later developed. This approach would require long-term partnerships with actors, and acknowledgement of the importance of tangible action during the research project which can change those involved along that journey. This is not automatically served by working with Non-Governmental organisations (NGOs). which demands that we imagine different methods that consider the what and how for working with communities and citizens at scale. In Colombia, we have done this by working within existing long-term partnerships. Using with shared decision-making and large-scale community involvement in our intervention design processes, through adapting theory of change workshops for large numbers of participants, as one example.

Co-owned research approaches could also take the shape of identifying citizens and communities’ own research priorities, and working with them towards their achievement in ways that are defined by communities themselves. There is precedent among Indigenous communities who set clear boundaries on the nature and forms of that research that can be conducted with their involvement. This is a direct response to histories of exploitative research practices, which promote research for the sake of research with little to no benefit for citizens involved. For example, the Southern African San People’s research code of conduct^21^ demands a transformative purpose to research that is aligned with their own vision for the future of their people. In the absence of such frameworks, ‘gold-standard’ global health work would seek to support community actors in the development of these structures to organise futureknowledge production processes which would position them as true equals.

As we inch towards a more equitable future in global health, tensions will always remain with how to grapple with the wake. There is surely nothing bigger than the ‘wake’ in which global health lives: the ever-widening gaps between the rich and poor, unbalanced distribution of the devastation of climate change and their undeniable links to our colonial histories. This is often made worse by the weight of the problematic archives to which our work is perpetually anchored. Perhaps by orienting our work towards transformation that explicitly aligns with the visions of those who live in the wake, we may find our best hope for a pragmatic way forward in the long term. Pragmatic because it does not assume that this change happens all at once; necessary because always it is turned towards citizens. Transformative paradigms do not ‘call’ for decolonisation. They just go about the work of identifying ways to respond to it; by letting go of multiple forms of power held for too long.

Not every project will achieve ‘the dream’ but if our basic foundations are co-constructed questions built with/by communities, rather than simply a desire to contribute to our archive of evidence—who knows what direction global health could take?

Perhaps that is now where our energies must lie; in the slow steps to build real structures that eventually mean an end to the need for a ‘global health’ at all.
